# Rapid Gout Detection Method and Kit

**DOI:** 10.3390/diagnostics9040157

**Published:** 2019-10-22

**Authors:** Srinivas Pentyala, Rebecca Monastero, Sai Palati, Elizabeth Varghese, Amith Anugu, Sahana Pentyala, Vamiq M. Mustahsan, David E. Komatsu, James Penna, Lawrence C. Hurst

**Affiliations:** 1 Department of Anesthesiology, Stony Brook University, Stony Brook, NY 11794, USA; Rebecca.Monastero@stonybrook.edu (R.M.); Sai.Palati@stonybrook.edu (S.P.); elizabeth.j.varghese@stonybrook.edu (E.V.); Amith.Anugu@stonybrook.edu (A.A.); vamiqmohammed.snu@stonybrook.edu (V.M.M.); 2 Department of Health Sciences, Stony Brook University, Stony Brook, NY 11794, USA; 3 Department of Urology, Stony Brook University, Stony Brook, NY 11794, USA; 4 Department of Physiology & Biophysics School of Medicine, Stony Brook University, Stony Brook, NY 11794, USA; 5 Department of Orthopedics, Stony Brook University, Stony Brook, NY 11794, USA; David.Komatsu@stonybrook.edu (D.E.K.); James.Penna@stonybrook.edu (J.P.); Lawrence.Hurst@stonybrook.edu (L.C.H.)

**Keywords:** gout, diagnostic, biomarkers, monosodium urate crystals, point-of-care

## Abstract

Gout is a form of arthritis characterized by buildup of uric acid in synovial fluid, which causes severe swelling and can harm joints, tendons, and other tissues. It affects approximately 4% of the United States population, or approximately 8.3 million people nationwide and is therefore a topic of epidemiologic consideration due to its prevalence. Gout is typically diagnosed via polarized microscopy of arthroscopically-aspirated synovial fluid, which is a costly, time-consuming, labor-intensive, and technically complex procedure, warranting a simpler and less complex method for diagnosis. Here, we propose and validate a colorimetric method which is based on the ability of uric acid to reduce silver nitrate. We also assessed how the colorimetric change can be accelerated by changing the concentration of silver nitrate or adding different silver catalysts, as well as develop a matrix bed for improved handling and ease of use. When translated to the clinic, this diagnostic method for gout will have the potential to increase diagnostic efficiency and accelerate patient care at the bedside.

## 1. Introduction

Gout is a form of arthritis characterized by the buildup of urate crystals in synovial fluid, which can cause swelling, joint pain, and damage to tissues. The disease is relatively common, affecting approximately 4% of the US population, and factors which may contribute to its presentation include dietary animal purines, alcohol, fructose, obesity, diuretic use, and metabolic syndromes [1]. While the age-adjusted prevalence of gout and hyperuricaemia has remained unchanged in the most recent decade from 2007–2008 to 2015–2016 in US, the estimated total number of persons with self-reported gout has increased from 8.3 million to 9.2 million [2].

Serum urate levels are influenced by genetic and environmental factors and the interrelation between these two [3]. The elimination of milk or other dairy products due to cow milk allergies might increase the risk for gout and hyperuricemia [4]. Early-onset hyperuricemia has been shown to have strong genetic components [5]. Urate exporter gene (*ABCG2*) variants have been shown to increase the risk of hyperuricemia [6] and the most common dysfunctional *ABCG2* variant—*rs2231142a*—has been reported to significantly influence the age of onset of gout and is highly associated with familial gout history [7]. *ABCG2* dysfunction was also reported to be a strong independent risk factor for pediatric-onset hyperuricemia [8].

**Figure 1:**
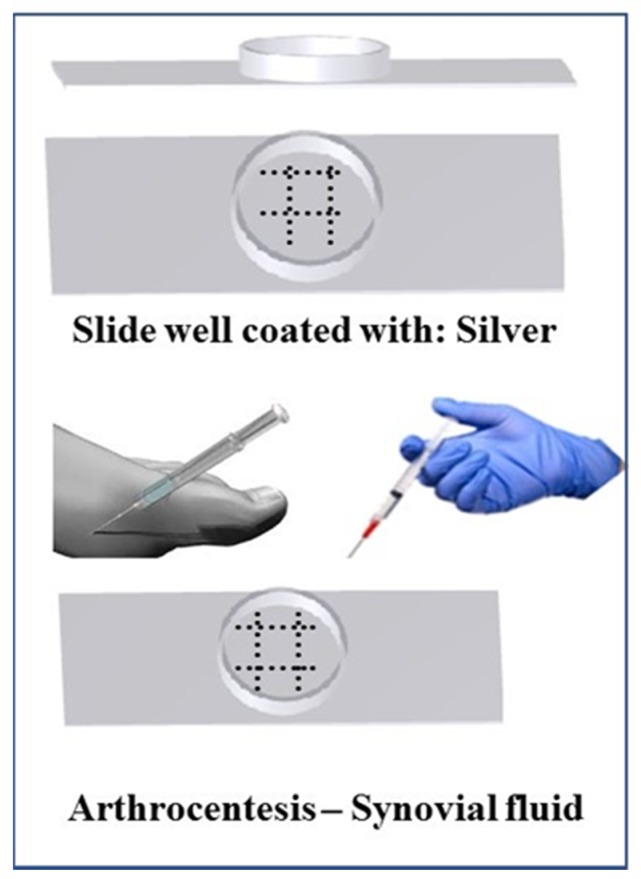
Rapid gout detection device prototype. The device consists of a leak-proof round well of 1.5 mm diameter on a plastic slide. The base of the well will be coated with reactive-ready silver nitrate solution and incubated overnight in a dehydrator to have dried layer of silver nitrate on the base.

The standard diagnostic test for gout is the identification of monosodium urate crystals in synovial fluid. Identification of monosodium urate crystals cannot be done visually by the naked eye, and therefore uses the well-established technique of polarized microscopy on joint fluid obtained through arthrocentesis. However, this microscopy technique is a time-intensive and costly technique which requires trained technicians, and there is a higher probability of false negatives appearing due to crystal dissolution if an extended timeframe is required to process the samples. Further, urate crystals are only identified correctly 81% of the time even when examined by trained technicians and rheumatologists [9]. Therefore, we developed a less expensive, accurate, faster, and less technically challenging detection method and device (Figure 1) with greater sensitivity, where synovial fluid can directly be tested bedside for the presence of gout. Currently, no point-of-care or bedside techniques are available to detect gout, and thus a bedside gout detection technique is necessary to improve efficiency of patient care.

A similar condition to gout is known as pseudogout, which is characterized by precipitation of calcium pyrophosphate crystals in synovial fluid. Pseudogout can cause swelling, tenderness, and pain in the joints like that of gout [10]. Whereas gout usually occurs in the metatarsal joint of the first toe, tarsal joints, ankle, or knee, pseudogout most often occurs in the knee [11]. Due to the similarities of the presentations of gout and pseudogout, it is vital that any diagnostic method for gout be able to distinguish between urate and calcium phosphate crystals to ensure a proper diagnosis and subsequent treatment.

Another diagnostic distinction that is important to make when evaluating arthralgia clinically, is differentiating between septic arthritis and gouty arthritis. Septic arthritis can present similarly to gout, in that, both present with acute monoarthritis. Septic arthritis is much more serious clinically and requires immediate surgical intervention. There are a variety of clinical and laboratory variables that may be used to predict the probability of gout versus septic arthritis, though these are not always correct in distinguishing the two; the only way to surely rule out a diagnosis of gout is through analysis of synovial fluid [12]. Therefore, a bedside test for gout is warranted to improve distinction from septic arthritis.

Currently, various uric acid detectors are on the market, including EasyTouch^®^, UASure, HumaSens, and Benecheck. However, these do not necessarily diagnose gout, as elevated uric acid levels in blood do not always indicate gout, but rather identify the potential for gout or a gout flare-up following initial diagnosis. Therefore, these tools do not have the potential to establish the standard-of-care. Further, these spectroscopic methods require repeat testing, and the arthralgia associated with gout means a comparatively more rapid method is indicated. A Raman-based spectroscopic tool was developed recently, but it too has limitations: the tool does not have the ability to distinguish between gout and pseudogout and it is ergonomically impractical [13]. Thus, there is currently no tool on the market which can diagnose gout accurately, quickly, and inexpensively at the bedside, warranting development of such a device.

## 2. Methods

### 2.1. Preparation of Urate Crystals

Methods to prepare urate crystals of size 5–25 micrometers were based on the protocols described by McCarty and Faires, as well as Mandel [14,15]. For urate crystal preparation, 1.68 g of uric acid amorphous powder was dissolved slowly in 400 mL of distilled water at 60 °C while stirring; 0.45 g of NaOH was then added to the solution. To result in a final solution pH of 7.0, 1N HCl was added as necessary. The solution was cooled for 48 h at 23 °C. Crystal form in the suspension was harvested and verified using microscopic examination.

### 2.2. Urate Detection: Silver Nitrate Solution

Silver nitrate is reduced to silver and turns black upon addition of uric acid, which is the colorimetric basis for our rapid uric acid detection method. Urate crystals were added to glass wells of a 24-well cell culture dish with 200 microliters of varying concentrations of silver nitrate solution: 100%, 80%, 60%, 40%, 20%, and 10% silver nitrate. The culture plate was incubated at room temperature for 30 min and images were taken. All imaging was completed via a Proscope Micromobile microscope attached to an iPhone. Compared to other concentrations, 20% silver nitrate showed the most effective staining upon urate addition; therefore 20% silver nitrate solution was used in subsequent experiments when silver nitrate concentration was not variable.

### 2.3. Urate Detection: Pre-Coated Staining Agent

Varying concentrations (10%, 20%, 40%, 60%, 80%, and 100%) of silver nitrate solution were added separately to glass wells in a 24-well cell culture dish and allowed to dry overnight. Excess solution was removed after 24 h. Amorphous uric acid powder (1 mg/200 µL) suspension was prepared in saline and 1.0 mL was added to the wells coated in dried silver nitrate. Wells were incubated at room temperature for 30 min and images were taken. Compared to other concentrations, 20% silver nitrate coating showed the most effective staining upon addition of urate.

### 2.4. Distinguishing Urate Crystals from Calcium Crystals

To rule out a diagnosis of pseudogout, it is necessary to confirm that calcium pyrophosphate crystals, which are characteristic of pseudogout, are not present. This distinction can be made using alizarin red solution (1%), which interacts with calcium pyrophosphate crystals, but does not interact with urate crystals. Accordingly, if alizarin red does not cause a colorimetric change upon addition of a solution, though silver nitrate does so, calcium pyrophosphate crystals are not present and urate crystals are present, which supports a diagnosis of gout. We demonstrated this by adding urate crystals to both a 20% silver nitrate solution and 1% alizarin red; a colorimetric change was observed in the wells with silver nitrate, but no change was noted upon addition of urate to the solution of alizarin red.

### 2.5. Ammoniacal Silver Stain

Ammoniacal silver stain for catalyzed silver reduction was prepared according to a modified version of the protocol of Sinha et al. (2001). Stock solutions for the ammoniacal silver stain were prepared according to the following: (a) Silver nitrate stock solution (1.14 M): 3.873 g of silver nitrate in 20 mL distilled water; (b) NaOH (90 mM): 0.18 g NaOH in 50 mL water; and (c) citric acid (47.6 mM): 0.5 g of citric acid in 50 mL water. Developer stock solution of formaldehyde was prepared to 12 mL total volume by adding 0.009 mL citric acid stock and 0.15 mL of 4% formaldehyde to 11.841 mL water. To prepare the ammoniacal silver stain of 15 mL, 3.15 mL NaOH stock was added to 11.0 mL of water, and then 0.175 mL of concentrated ammonium hydroxide was added dropwise. Silver nitrate was added dropwise (0.6 mL) to the solution to total 15 mL of ammoniacal silver stain solution.

### 2.6. Nondiamine Silver Stain

Nondiamine silver stain for catalyzed silver reduction was prepared according to Sevchenko et al. [16]. To prepare the stain, 0.15 mL of 4% formaldehyde and 0.36 g of sodium carbonate were added to 11.85 mL of distilled water.

### 2.7. Urate Crystal Detection: Catalyzed

Two six-well plates were coated with ammoniacal silver stain and nondiamine silver stain, respectively, to determine their effectiveness of catalysis. Silver nitrate solution of 0.5 mL was added to the wells on one plate and then removed after 6 h to dry. Stains were left to dry at 23 °C for 24 h. Reagents listed below were then added in volumes of 0.5 mL to the stained wells, with urate crystal suspension added last. Images were taken of the colorimetric reaction before urate was added, immediately after urate was added, and every 5 min after the addition of urate up to 20 min elapsed. Plate one reagents (non-diamine silver stain) included: (a) well 1: urate; (b) well 2: urate, formaldehyde; (c) well 3: urate, bicarbonate; (d) well 4: formaldehyde, bicarbonate; (e) wells 5 and 6: urate, formaldehyde, bicarbonate. Plate two reagents (ammoniacal silver stain) included: (a) well 1: urate; (b) well 2: urate, formaldehyde; (c) well 3: urate, citric acid; (d) well 4: formaldehyde, citric acid; (e) wells 5 and 6: urate, formaldehyde, citric acid.

### 2.8. Urate Crystal Detection: 20% vs. 1.14 M

To prepare 20% silver nitrate solution, 2 mL of 100% nitrate was combined with 8 mL distilled water. To prepare 1.14 M silver nitrate solution, 3.783 g of silver nitrate powder was added to 20 mL water. Three wells (wells 1–3) of a six-well plate were coated with 20% silver nitrate solution, and the remaining three wells (wells 4–6) were coated with 1.14 M silver nitrate solution. At the following specified times, 0.5 mL of urate crystal solution was added to the wells: images were taken before urate was added, immediately after urate was added, and every 5 min after addition of urate up to 20 min elapsed.

Two of the wells (1 and 4, 20% and 1.14 M silver nitrate solutions, respectively) were labeled “wet condition:” urate crystal solution was added while silver nitrate solutions remained in the wells; silver nitrate solution was not removed prior to urate crystal suspension addition. Two wells (2 and 5, 20% and 1.14 M silver nitrate solutions, respectively) were labeled “wet/dry condition:” urate crystal suspension was added immediately after removal of silver nitrate solution. The remaining two wells (3 and 6, 20% and 1.14 M silver nitrate solutions, respectively) were labeled “dry condition:” urate crystal solution was added after the silver nitrate stain had been in the well for 6 h and had then dried for 24 h.

### 2.9. Urate Crystal Detection: Dry Stain

Silver nitrate staining solutions were prepared of varying concentrations via dilutions of 100% stock silver nitrate solution. Concentrations of solutions prepared included: 10%, 20%, 40%, 60%, 80%, 100%. Each silver nitrate staining solution was added to an individual well on a six-well plate in a volume of 1.0 mL. All silver nitrate solutions were removed from the wells after 6 h and left to dry at 23 °C for 24 h. Urate crystal suspension in a volume of 1.0 mL was added to all wells once the stain had dried. Images were taken before urate was added, immediately after urate was added, and every 5 min after the addition of urate up to 20 min elapsed.

### 2.10. Matrix Bed Development

For development of immobile detection matrices, 6% gelatin mixture was mixed with 20% silver nitrate and added to wells, and 1.2% agar was mixed with 20% silver nitrate and added to a separate well plate. Polymerization solution (0.5 mL) was added to both the gelatin/silver nitrate and agar/silver nitrate wells. The mixture was air-dried for 24 h. Urate crystals and urate crystal suspension (1 mg/200 microliters) in saline solution was added to individual dried well matrices and images were acquired upon reaction.

## 3. Results

**Figure 2:**
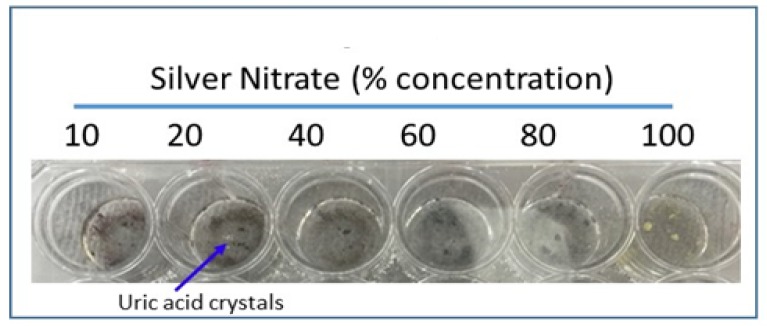
Reduction of silver nitrate solution of increasing concentrations by crystallized urate. Urate crystals were added to 200 µL solutions of increasing concentration of silver nitrate; 20% silver nitrate solution demonstrated most effective staining.

**Figure 3:**
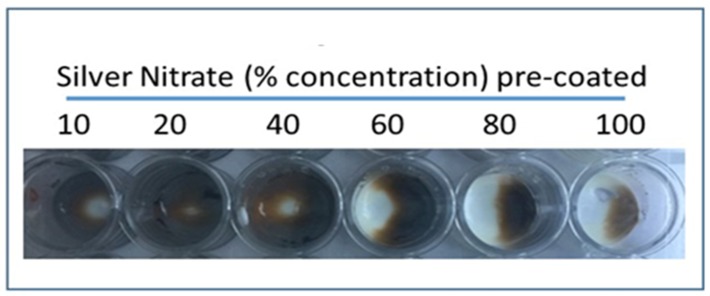
Reduction of silver nitrate pre-coated stains of increasing concentrations by crystallized urate. Varying concentrations of silver nitrate solution were added to wells and allowed to dry overnight, before being removed by pipet. Amorphous uric acid powder (1 mg/200 µL) in suspension was added to each of the wells; 20% silver nitrate showed most effective staining.

**Figure 4:**
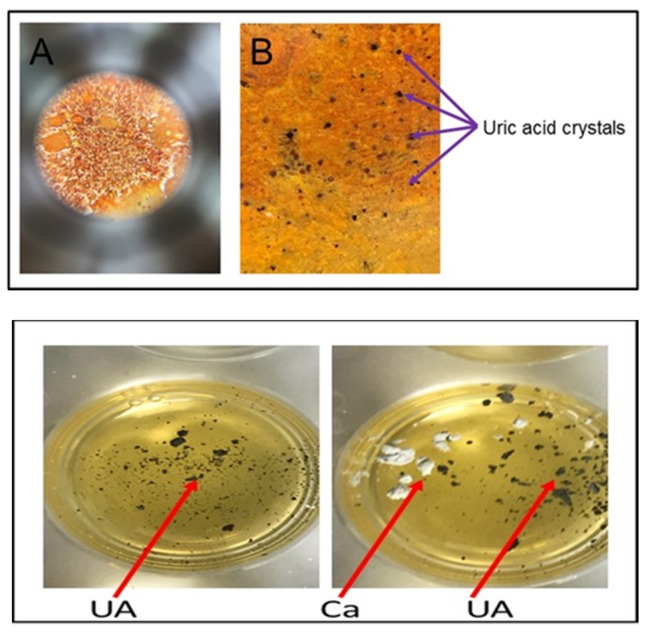
Distinguish between urate and calcium crystals. Panel 1: Urate crystals were added to stain (20% silver nitrate and 1% alizarin red) pre-coated gout detection device. Images were taken with Iphone-6 attached with Proscope Micromobile microscope (no polarizing filter was used). A: 1× magnification; B: 10× magnification. Urate crystals are stained black. Panel 2: Urate and Calcium pyrophosphate crystals were added to 20% silver nitrate—gelatin bed. Urate crystals are stained black whereas Calcium pyrophosphate crystals are unstained.

Most effective staining with amorphous uric acid powder was shown with a 20% concentrated silver nitrate solution, among other tested concentrations (Figure 2 and Figure 3). These results were consistent between wells of aqueous silver nitrate solution and dried, stained silver nitrate. Alizarin red solution (1%) was used in ruling out interference with calcium pyrophosphate crystals as urate does not react with alizarin (Figure 4).

**Figure 5:**
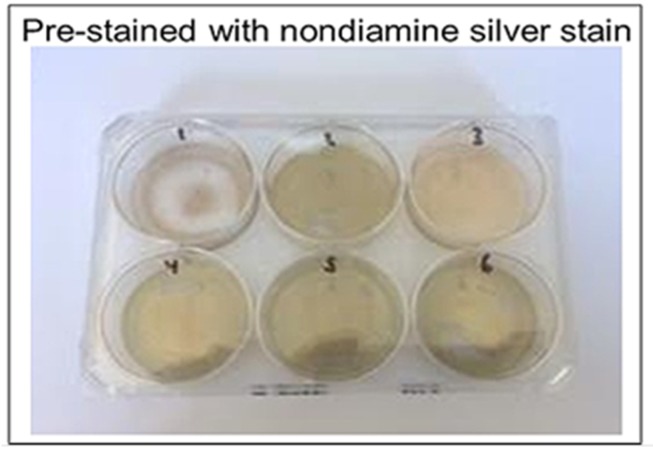
Nondiamine silver stain. This figure illustrates the effectiveness of nondiamine silver staining in catalyzing silver reduction by urate. Well reagents are as follows: 1 = urate; 2 = urate, formaldehyde; 3 = urate, sodium bicarbonate; 4 = formaldehyde, sodium bicarbonate; 5 = urate, formaldehyde, sodium bicarbonate; 6 = urate, formaldehyde, sodium bicarbonate.

**Figure 6:**
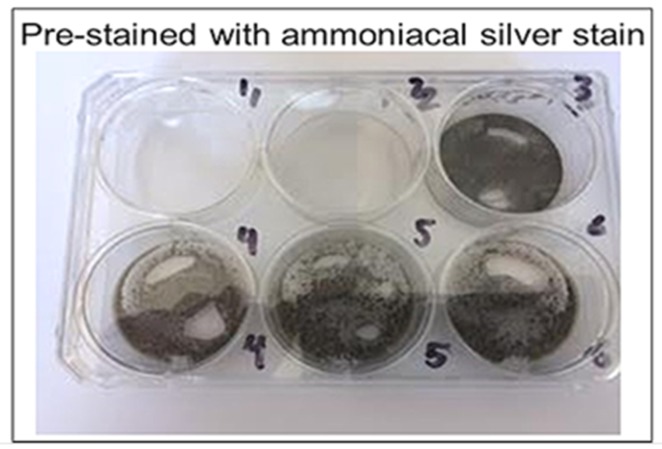
Ammoniacal silver stain. This figure illustrates the effectiveness of ammoniacal silver staining in catalyzing silver reduction by urate. Well reagents are as follows: 1 = urate; 2 = urate, formaldehyde; 3 = urate, citric acid; 4 = formaldehyde, citric acid; 5 = urate, formaldehyde, citric acid; 6 = urate, formaldehyde, citric acid.

Ammoniacal silver showed relatively faster reduction of silver when compared to the non-catalyzed silver solution and stain, as well as compared to the nondiamine stain (Figure 5 and Figure 6). The ammoniacal silver stain showed visually discernable colorimetric change after 10 min, whereas the nondiamine silver stain did not show such a change after the same amount of time. The nondiamine stain did not show significant colorimetric change until after 20 min had elapsed. These results indicate greater potential catalytic utility of the ammoniacal silver stain when compared to the nondiamine stain.

When comparing 20% silver nitrate solution to the 1.14 M solution, colorimetric results were similar between groups except for the completely dry stain. All wells were completely clear of color to the naked eye before addition of urate. With wells 1 and 4, as well as 2 and 5, the 20% silver nitrate solution showed similar results, turning black in a similar timeframe when compared to the 1.14 M solution. The 20% solution and 1.14 M solution both showed a colorimetric change at approximately 5 min for wells 1 and 4, and both showed a colorimetric change between 10 and 15 min for wells 2 and 5. However, for wells 3 and 6, which contained the completely dry stain before urate addition, the 20% silver nitrate stain was considerably more effective in showing a colorimetric change at an earlier block of time (Figure 5). The dried 20% silver nitrate stain (well 3) showed a colorimetric change between 5 and 10 min, whereas the dried 1.14 M silver nitrate stain (well 6) did not show a considerable colorimetric change until approximately 15 min (Figure 6).

**Figure 7:**
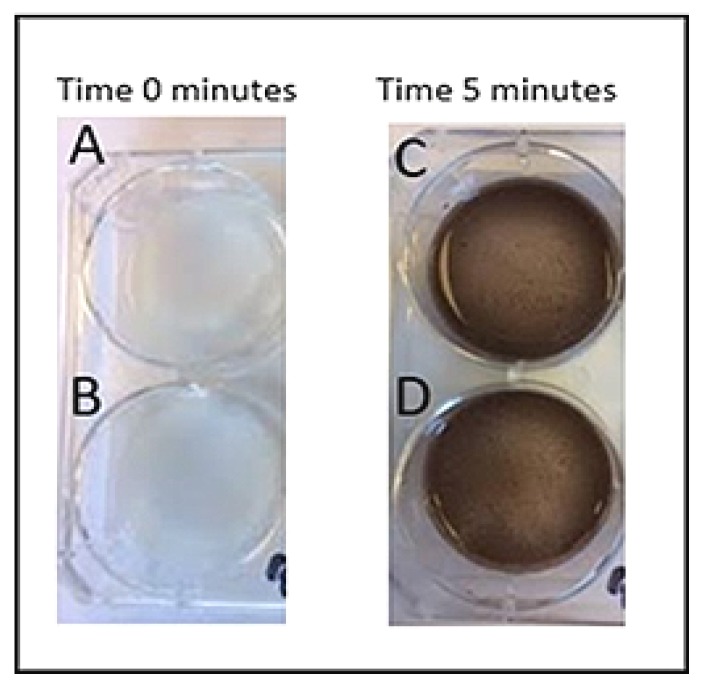
Comparison of 20% silver nitrate solution and 1.14 M silver nitrate solution upon addition of urate. Top photos: 20% silver nitrate; bottom photos: 1.14 M silver nitrate solution. First column of photos: 0 min elapsed; second column of photos: 5 min elapsed.

When comparing different concentrations of dried silver nitrate to detect crystallized urate, silver nitrate stain concentrations greater than 20% were most effective colorimetrically. Although detecting amorphous uric acid powder was optimal at 20% silver nitrate concentration, urate crystals were best detected at higher concentrations of silver nitrate stain. Higher concentrations of silver nitrate stain were colorimetrically effective within 5 min, and lower concentrations were effective between 10–15 min (Figure 7).

**Figure 8:**
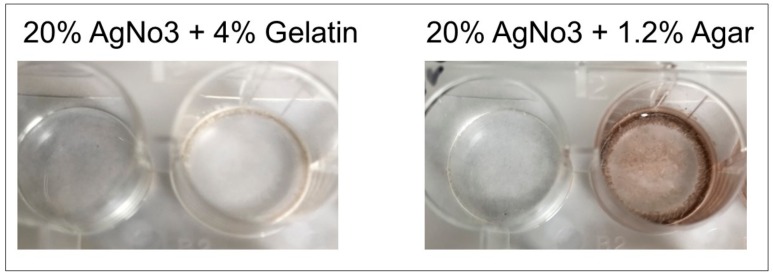
Matrix bed development with 6% gelatin or 1.2% agar. Left image: 6% gelatin was mixed with 20% silver nitrate and added to wells, followed by polymerization solution (0.5 mL); right image: 1.2% agar was mixed with 20% silver nitrate and added to wells, followed by polymerization solution (0.5 mL). Upon development of the agar matrix, a color was produced, and therefore gelatin matrices will be preferentially developed for colorimetric reaction.

**Figure 9:**
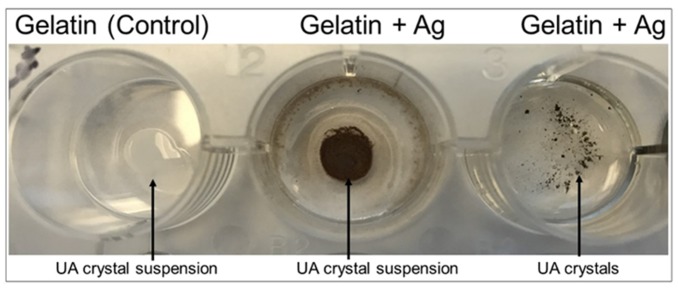
Gelatin matrix testing with urate. Gelatin-silver nitrate beds were developed as previously discussed. Urate crystal suspension and dried urate crystals were individually added to the matrix beds. Both the crystals and crystal suspension induced an effective colorimetric change upon addition to the gelatin-silver nitrate matrix.

Matrix beds were successfully developed and will be used in the clinical product. Due to the dark color produced upon the formation of the agar-silver nitrate matrix (Figure 8) which would interfere with our colorimetric method, the gelatin-silver nitrate matrix beds will be utilized preferentially. Both urate crystals and a crystal suspension produced effective colorimetric results in the gelatin-silver nitrate matrix beds, validating the gelatin matrices’ use in future product development (Figure 9).

## 4. Discussion

In most developed countries, gout affects over one percent of the population; in the United States specifically, gout affects over 3.9% of the population [17]. The incidence and prevalence of gout has been rising in recent decades, increasing the global burden of the disease [18]. Globally, gout incidence ranges from 0.06 to 2.68 per 1,000 person-years [19]. Due to such high gout incidence and prevalence in the United States and globally, the market for gout detection devices in hospitals and clinics may accordingly increase. Medical specialists who typically diagnose gout include primary care physicians, rheumatologists, and orthopedists, which means there are approximately 244,000 diagnosing United States physicians comprising the market for gout detection devices [20]. The market for these devices will also consist of the 5,564 registered hospitals in the United States where rapid gout detection is warranted [21]. Therefore, due to the high and rising prevalence of gout and the large number of physicians and hospitals with the capacity to diagnose gout, there is a considerable market for such a diagnostic device.

The above results validate our silver nitrate-based bedside gout detection method, and additionally show that the test can distinguish between gout and pseudogout by testing also for the presence of calcium pyrophosphate crystals via alizarin red. Although 20% silver nitrate was optimal in detecting amorphous uric acid, testing showed that higher concentrations were more effective in causing a colorimetric change with crystallized urate. This suggests that higher concentrations should be used for the silver nitrate stains in a point-of-care method, as urate is present in crystal form in synovial fluid of gout patients and therefore these higher concentrations correlate with clinical utility. However, the exact concentration of silver nitrate stain for the bedside device will be evaluated further in studies using simulated synovial fluid and clinical studies using synovial fluid from patients. Nonetheless, these results validate the utility of the gout detection method and suggest possible concentrations for the silver nitrate stain to be used in the bedside device.

We note that an ammoniacal silver stain is more effective in colorimetric distinction of urate crystals when compared to nondiamine stains. However, higher concentrations of silver nitrate solution showed slightly faster colorimetric results. The slightly slower colorimetric change with the catalyst may be due to trace impurities in water used for dilution or fluctuations in temperature of reagents, despite attempts to control environmental factors. As per these results, higher concentrations of silver nitrate stains may indeed be more relevant to clinically applied methods, rather than catalyzed stains. Nonetheless, both ammoniacal-catalyzed and uncatalyzed silver nitrate stains provide colorimetric results within 5 to 10 min, indicating that both would be suitable choices for clinical product development.

These results demonstrate that our method is a novel way of identifying urate crystals bedside. Our novel test may be completed in less than five minutes based on our results, depending on the concentration of silver nitrate and presence of catalysts, and does not require trained technicians. Thus, our newly validated method is more efficient, less expensive, and less labor-intensive than current gout detection methods. Whereas spectroscopically-based methods such as EasyTouch^®^, UASure, Benecheck, and HumaSens require repeat attempts/use for measurement and only identify changing uric acid levels in blood, our method can be used for a first-time diagnosis of gout, rather than only for monitoring after an initial attack [22]. Our method also is more advantageous when compared to a recently developed Raman-based spectroscopic tool, as the tool is ergonomically impractical and does not distinguish between gout and pseudogout, whereas our method does not have such limitations [13]. Additionally, is important to recognize the potential for considerable sensitivity which this method may have upon clinical validation. Due to the colorimetric nature of the test, even notably small volumes of synovial fluid can be analyzed accurately to provide a diagnosis. Therefore, if there is difficulty with collection of synovial fluid, or if there is sparse urate crystal presence, this test will demonstrate the advantage of an accurately positive result despite these challenges. Our proposed rapid diagnostic method may shift the usage away from spectroscopic and microscopic methods of diagnosis, as it will be the first colorimetric gout detection method.

The speed of diagnosis via our gout detection method has implications for necessarily rapid pain management in gout patients. Gout causes severe physical impairment and disability during flare-ups, and even after an acute episode of a gout flare-up, the pain occurring during the acute episode usually does not subside considerably without medication [23]. Gout has a significant impact on a patient’s quality of life by disrupting patients’ sleep, increasing patients’ dependency on family and others, and preventing patients from carrying out daily recreational activities, among other flare-up effects [24]. Due to the high pain burden of this disease, the speed of diagnosis is critical to relieving patients’ discomfort through the prompt prescription of a pharmacologic agent. Polarized light microscopy typically takes approximately two hours, if there is an in-house pathology laboratory, but if the sample must be sent out for analysis, processing can take up to two days, which limits the speed of diagnosis and treatment. Our test can diagnose gout in less than ten minutes, which provides an excellent timeframe for management of the disease to best relieve patients’ pain when compared to the standard-of-care. However, there are certain limitations to this rapid gout detection method. Being a point-of-care diagnostic method, the results obtained from this test will not differentiate other type of monoarticular arthritis like gonococcal or Lyme arthritis. However, our test can readily differentiate between gout, pseudogout and infection in synovial fluid, a dilemma that is encountered by a clinician when a patient presents with symptoms that may appear to be gout. Also, this point-of care test will provide a positive or negative diagnosis and will not be able to diagnose between mild vs moderate vs severe gout. This test readily identifies the presence of urate crystals in synovial fluid there by providing the physician to plan for treatment of gout immediately.

The speed, accuracy, and ease-of-use of this diagnostic method has implications in gout detection not only in the United States health care system, but also in less developed countries with limited access to health care, particularly because the detection method is easily used and transported and can also be used without technical training. Pending clinical validation, this method will translate as the first bedside detection kit for the diagnosis of gout, which will permit expedited and cost-efficient diagnoses.
